# Reduction of sulfur in fuel oil using Fe_2_O_3_ hybrid nanoadsorbent by solvent deasphalting and optimization of operational parameters with CCD

**DOI:** 10.1038/s41598-024-52166-5

**Published:** 2024-01-18

**Authors:** Mohammadreza Malek, Mohammad Samipourgiri, Alimorad Rashidi, Nasrolah Majidian

**Affiliations:** 1 Chemical Engineering Department, Faculty of Engineering, Islamic Azad University, North Tehran Branch, Tehran, 1651153311 Iran; 2 Carbon and Nanotechnology Research Center, Research Institute of Petroleum Industry (RIPI), Tehran, 14857-33111 Iran

**Keywords:** Chemical engineering, Nanoscience and technology

## Abstract

The present study investigated and tested the effect of adding three types of nanoadsorbents (multi-walled carbon nanotubes (MWCNT)) in pure form, multi-walled carbon nanotubes with Fe_2_O_3_ particles (MWCNT-Fe_2_O_3_) hybrid, and Silanated-Fe_2_O_3_ hybrid to heavy fuel oil to reduce sulfur using a deasphalting process with solvent. First, all three types of nanoadsorbents were synthesized. Then, the Central Composite Design (CCD) method was used to identify the parameters effective in deasphalting, such as the type of nanoadsorbent, the weight percentage of nanoadsorbent, and the solvent-to-fuel ratio, and to obtain their optimal values. Based on the optimization result, under laboratory temperature and pressure conditions, the highest percentage of sulfur reduction in deasphalted fuel (DAO) was obtained by adding 2.5% by weight of silanated-Fe_2_O_3_ nano-adsorbent and with a solvent-to-fuel ratio of 7.7 (The weight percentage of sulfur in DAO decreased from 3.5% by weight to 2.46%, indicating a decrease of 30%). Additionally, by increasing the temperature to 70 °C, in optimal conditions, the results revealed that the remaining sulfur percentage in DAO decreased to 2.13% by weight, indicating a decrease of 40%. Synthesized nanoadsorbents and asphaltene particles adsorbed on the surfaces of nanoadsorbents were evaluated by XRD, FTIR, FESEM, and TEM techniques.

## Introduction

The global energy landscape is poised for substantial expansion, with projections indicating a roughly 50% surge in energy demand by the year 2040, amounting to an estimated daily consumption of around 400 million barrels of oil equivalent (mboe/d)^1^. This robust growth underscores the significance of nonconventional hydrocarbon sources, specifically heavy oil (HO) and extra-heavy oil (EHO). These unconventional reserves are particularly noteworthy, constituting nearly 70% of the world's total oil reserves^2^, with EHO reserves alone accounting for approximately 32% of the global oil reserves^3^. As the world's energy requirements continue to climb, these nonconventional resources emerge as pivotal contributors to the sustainable supply of hydrocarbons.

In the realm of modern energy production and the pursuit of sustainable fuel solutions, the refining of heavy crude oil remains a pivotal challenge. Amidst the myriad of processes and techniques employed in the petroleum industry, Solvent Deasphalting (SDA) emerges as a key player in the quest for high-quality fuel oils. Asphaltenes, the heaviest and most complex components of crude oil, pose significant operational challenges, including the formation of deposits, increased viscosity, and decreased product quality. To address these issues, and to enhance the efficiency and quality of fuel oil production, researchers and engineers have turned to a revolutionary approach the incorporation of nanoparticles into the Solvent Deasphalting process.

Solvent Deasphalting (SDA) technology utilizes light hydrocarbon alkanes, such as propane, butane, pentane, heptane, or naphtha, as solvents to facilitate the separation of crude oil into two distinct phases. These phases consist of the asphaltene-lean fraction, referred to as deasphalted oil (DAO), and the asphaltene-rich component known as pitch^4^. Several widely recognized SDA technologies have been developed, including the ROSE (Residuum Oil Supercritical Extraction by KBR), LEDA (Low Energy Deasphalting by Foster Wheeler), Demex technologies by UOP/Foster Wheeler, and the Solvahl process by IFP (France)/Axens^5–10^. These technologies represent pivotal contributions to the field, each offering distinct approaches to the vital process of solvent deasphalting.

The absorption of suspended asphaltene particles by different absorbents is one of the methods of separating and removing asphaltene and thus reducing the amount of sulfur in petroleum compounds. Some of these absorbents are nanoparticles, nanocomposites, zeolites, etc. In a study by Ansari et al.^11^, they presented the asphaltene absorption model on iron nano oxide (magnetite and hematite) and lime nanoparticles (calcite and dolomite). They found that the absorption power of asphaltene particles on the surface of iron nano oxide is higher than that of asphaltene particles on the surface of lime nanoabsorbents. Also, another study by H.Abbas et al.^12^, confirmed that the use of γ-Fe_2_O_3_ as a nano-absorbent in the removal of asphaltene particles in toluene solution and petroleum composition was very effective and achieved a high yield. Other valuable studies conducted in this regard investigated asphaltene particle absorption by environmentally friendly nanocomposites based on NiO^13–16^. These studies revealed that NiO/ZSM-5, NiO/SAPO-5, and NiO/AIPO-5 bio-nanocomposites in optimal laboratory conditions have removal power of asphaltene particles up to 90%, 88.02%, and 84%, respectively, indicating a very high potential in this field. The use of carbonaceous nanostructured compounds has also been investigated in asphaltene absorption from petroleum compounds in recent years. In one of these studies, Mansoori et al.^17^ examined the absorption effect of asphaltene with carbon nanostructured compounds such as graphene, multi-walled nanotube, black carbon, and activated carbon. They found that the highest absorption power is related to graphene, multi-walled carbon nanotubes, black carbon, and finally activated carbon, respectively. They also reported that one of the most significant factors for the absorption of carbon-based materials such as carbon nanotubes and graphene is the presence of strong π-π bonds between carbon nanostructures and asphaltene surface.

In the realm of solvent deasphalting, recent literature has introduced innovative approaches involving the incorporation of both macro- and microadsorbent materials. These techniques have garnered attention due to their documented success in enhancing both the yield and quality of deasphalted oil (DAO)^18,19^. Notably, several patents have also highlighted the utility of microscale solids, including clay, silica, alumina, and zeolite materials, to achieve superior quality in the DAO^20,21^.

However, it is noteworthy that, thus far, scientific literature has remained devoid of studies delving into the influence of nanoparticles (NPs) on the separation efficiency of the SDA process. The intriguing potential of nanomaterials in this context has yet to be explored extensively. Nanoparticles possess distinctive characteristics, including an impressive surface area-to-volume ratio, rendering them as host to a multitude of active sites. These unique attributes position them to play a pivotal role in selectively adsorbing asphaltenes onto their surfaces, thereby augmenting the extraction of asphaltenes^22–28^. The uncharted territory of nanoparticle integration into SDA heralds exciting prospects for refining the efficiency and efficacy of this process.

Shakir et al.^29^ conducted a pioneering study that harnessed the potential of Nanosilica for the enhancement of Iraqi heavy crude oil through the application of both solvent deasphalting (SDA) and enhanced solvent deasphalting (e-SDA) methodologies. The findings of this research revealed the remarkable impact of Nanosilica, as the optimal upgrading of heavy crude oil was achieved with the incorporation of 7 wt% NS. This transformation resulted in a significant increase in the API gravity to 35.9, accompanied by a remarkable 87.22% reduction in asphaltene content. Furthermore, the study demonstrated substantial improvements in the removal of sulfur, vanadium, and nickel by 51.17%, 55.07%, and 69.87%, respectively.

Ongarbayev et al.^30^ conducted a groundbreaking study involving the utilization of a zeolite adsorbent embedded with vanadium xerogel for the simultaneous demetallization and desulfurization of heavy oil residues. This innovative approach yielded impressive results, with a remarkable 90% extraction rate for vanadium, 70% for nickel, and 60% for iron. Moreover, the sulfur content exhibited a substantial reduction, decreasing from 1.97% to a significantly cleaner 1.36%. This research underscores the potential of zeolite-based adsorbents as a highly effective means of enhancing the quality of heavy oil residues while simultaneously reducing their environmental impact.

By introducing nanoparticles into the SDA process, it is possible to finely tune the separation of asphaltenes and other heavy components, resulting in cleaner and more valuable end products. This promising development not only promises to optimize the production of high-quality fuel oils but also offers the potential for reducing environmental impact and enhancing overall energy efficiency.

In the present investigation, we explore the impact of incorporating three distinct types of nanoadsorbents into heavy fuel oil to mitigate sulfur content via a solvent-based deasphalting process. These nanoadsorbents comprise multi-walled carbon nanotubes (MWCNT) in their pristine form, a hybrid configuration of multi-walled carbon nanotubes combined with Fe_2_O_3_ particles (MWCNT-Fe_2_O_3_), and a silanated-Fe_2_O_3_ hybrid. The objective is to examine how these nanomaterials influence the reduction of sulfur in heavy fuel oil through the deasphalting procedure.

## Materials and methods

### Synthesis and characterization of nanoadsorbent

To synthesize multi-walled carbon nanotubes (MWCNT), we employed the chemical vapor deposition (CVD) method with a CO-MO/MgO catalyst. The detailed procedure for MWCNT synthesis can be found in the study authored by Rashidi et al.^31^. For the creation of the MWCNT-Fe_2_O_3_ nanocomposite, we initiated the process by functionalizing carbon nanotubes through exposure to nitric acid (HNO_3_) under reflux conditions at 60 °C for a duration of 3 h. Subsequently, we dissolved 100 mg of FeCL_2_·4H_2_O in 30 ml of deionized water and ensured thorough mixing for 10 min using a magnetic stirrer. Then, 30 ml of the functionalized carbon nanotubes were added to the solution at room temperature. To regulate the pH, ammonia solution was gradually introduced into the solution, drop by drop.

**Figure 1:**
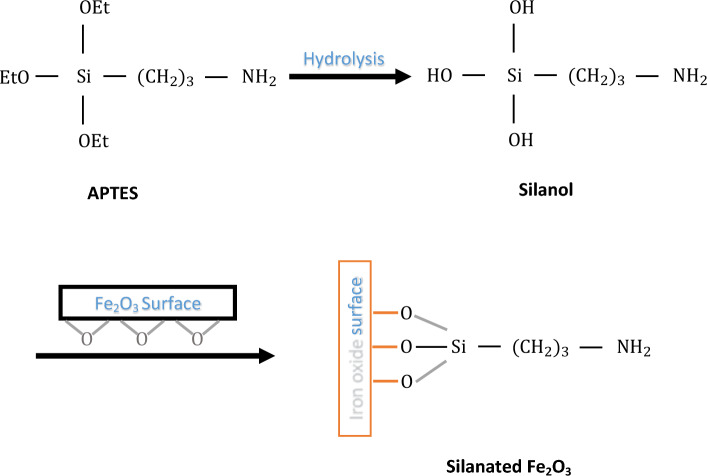
Steps of silanated-Fe_2_O_3_ synthesis.

Then, the prepared solution was heated for 2 h at a temperature of 80 °C and left alone to slowly reach the ambient temperature. Then, MWCNT-Fe_2_O_3_ particles were separated from the solution by centrifugation. The separated particles were washed several times with deionized water to reach a neutral pH. Finally, MWCNT-Fe_2_O_3_ nano-hybrid particles were dried in an oven at 80 °C for 6 h. For the synthesis of silanated-Fe_2_O_3_ nano-adsorbent, 0.5 g of Fe_2_O_3_ was dispersed in 400 ml of ethanol for 10 min using an ultrasonic bath. The chemical reaction of silanated-Fe_2_O_3_ particles was started by adding 2 ml of APTES substance, which was added drop by drop to the solution. Then, it was mixed with high speed for 24 h. Then, the Silanated-Fe_2_O_3_ nanoadsorbent was separated by a centrifuge and washed three times with an equal ratio of ethanol solution and deionized water. Finally, the Silanated-Fe_2_O_3_ nanoadsorbent particles were dried at 80 °C in the oven for 6 h. Figure 1 shows the steps of the synthesis of silanated-Fe_2_O_3_^32^. Three types of obtained nanostructures were evaluated and analyzed by XRD, FTIR, TEM, and FESEM techniques.

XRD (X-ray Diffraction) analysis is one of the widely used analyses in identifying compounds and phases in materials^33^. In this study, Philips pw1730 XRD was used for XRD analysis. FTIR (Fourier transform infrared spectrometer) analysis is one of the widely used analyses in identifying compounds and bonds in organic and inorganic materials^34^. In this study, FTIR Tensor ii was used for FTIR analysis. FESEM analysis is a member of the scanning electron microscope family. Its primary advantage over SEM analysis is its better resolution. This analysis is used to examine surface characteristics and morphology^35^. In this study, FESEM ZEISS sigma 300 was used for FESEM analysis. TEM analysis or transmission electron microscope is one of the most crucial analysis methods for studying materials and especially nanoadsorbents thanks to its extremely high capability in imaging particles at high magnifications. In this study, TEM Philips EM 208 s was used for TEM analysis^36^.

### Fuel oil sample

In this study, a fuel oil 380 sample from Bandar Abbas in the south of Iran was used as feed. Table 1 presents the characteristics of this fuel oil. Table 1 also shows the SARA analysis based on the ASTM-D2007 standard to detect the weight percentage of aromatics, saturates, resin, and asphaltene in fuel oil.

**Table 1:** Physical characteristics of fuel oil used in this study as a deasphalting process.

Test	Unit	Specification	Test method
Density @50 DC	Kg/m^3^	Max 990	ASTM D-1298
API		13.11	ASTM D-1298
Kinematic viscosity @50 DC	CST	Max 380	ASTM D-445
Sulfur total	Wt.%	Max 3.5	ASTM D-1552
Water	Wt.%	Max 1	ASTM D-1796
SARA analysis			
Saturates	Wt.%	21.40	ASTM D-2007
Aromatics	Wt.%	32.53	ASTM D-2007
Resins	Wt.%	28.99	ASTM D-2007
Asphaltenes	Wt.%	17.08	ASTM D-2007
Elemental analysis			
C	Wt.%	84.37	ASTM D-5291
N	Wt.%	0.81	ASTM D-5291
O	Wt.%	1.34	ASTM D-5291
H	Wt.%	9.98	ASTM D-5291

ASTM-D5291 standard was also used to determine the values of carbon, hydrogen, nitrogen, and oxygen elements in Fuel oil 380.

### Description of the experiment

In this study, deasphalting of fuel oil was done under the temperature and pressure conditions of the laboratory environment. In each step of the experiment, a certain amount of the desired nanoadsorbent was added to the normal heptane solvent with a certain volume ratio and it was ultrasonicated for 20 min. Then, 20 g of fuel oil was added to the solution and mixed in a mixer for 3 h with a magnetic magnet and at a speed of 700 rpm. After this step, the contents of the mixer were transferred to the special container of the centrifuge (Falcon). The amounts stuck to the sides of the balloon were washed again with normal heptane and transferred to the Falcons to prevent fuel loss. The falcons were kept standing for 24 h away from light, so the asphaltene particles suspended in the solution were adsorbed by the nanostructures and fell to the end of the falcons by gravitational force. In this step, asphaltene deposition was visible at the end of the falcons. In this step, the falcons were centrifuged at 8000 rpm for 15 min and the separation was done.

The DAO consisted of deasphalted fuel and normal heptane. The pitch consisting of the separated asphaltene along with the nanoadsorbent was collected as a layer of cake at the end of the container. The separated asphaltene was washed again with normal heptane to separate its non-asphaltenic compounds. Then, it was placed in an oven at a temperature of 65 °C for several hours to evaporate the small amount of solvent in it. After the mass amount of isolated asphaltene did not change over time, it was kept in a closed container for analysis. Pitch yield was calculated based on the following Eq. (1):

1 $$pitch \;yeild \left(\% Wt \right)= \frac{weight \;of \;dried \;pitch (gr)}{Fuel \;oil \left(gr\right)}\times 100$$

**Figure 2:**
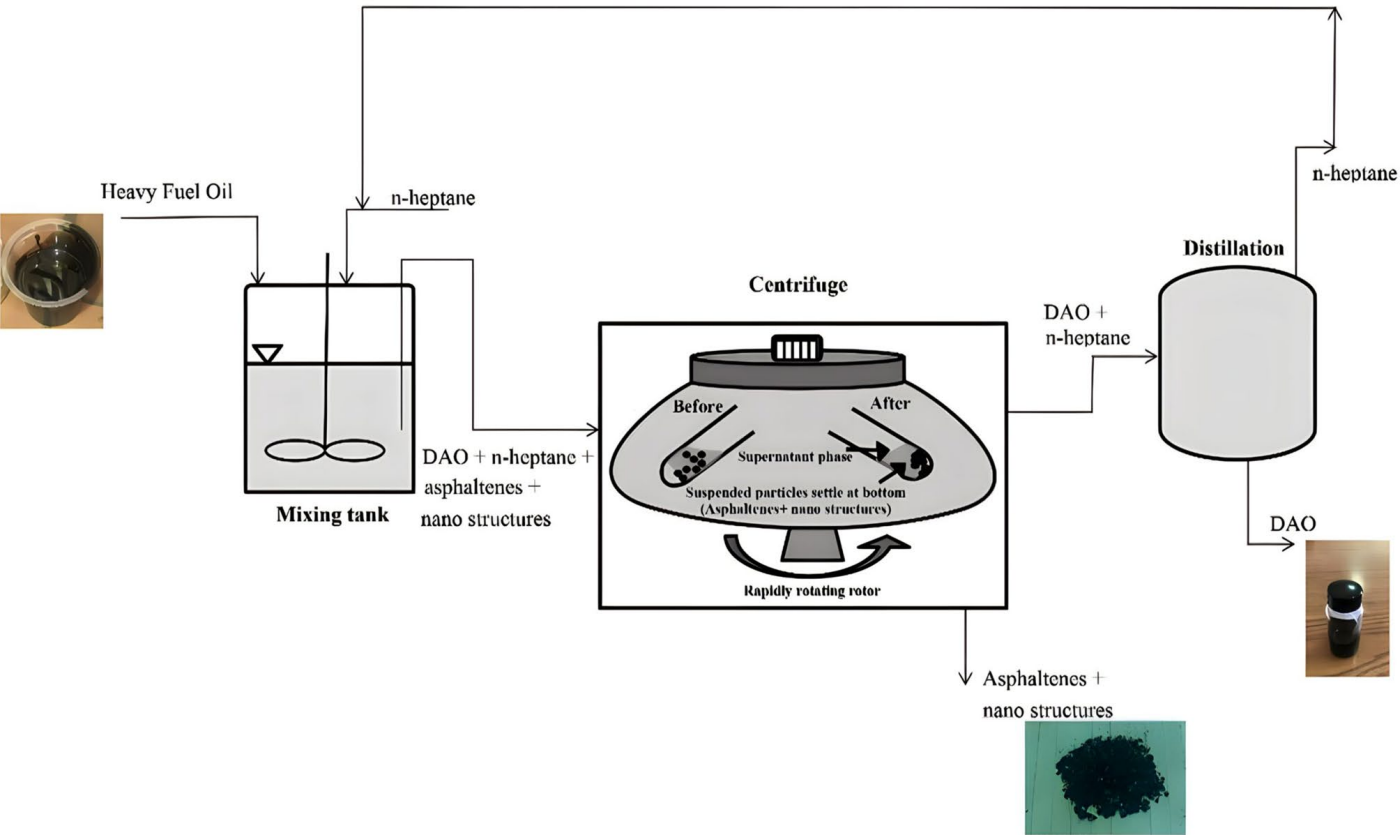
The schematic of the deasphalting process of fuel oil used in this study.

In each step, the solvent in DAO was separated by a distillation system and stored in a container for reuse. The amount of solvent-free DAO sulfur was measured by standard D-1552. The isolated asphaltene also underwent XRD, FTIR, and FESEM analysis for further examination. Figure 2 shows the schematic of the process used in this study.

### Experiment design

In this study, Design Expert software (version 13.0.5.0) was used to design the experiment. Generally, experiment design and response surface method (RSM) can be used as a powerful tool for effective and maximum utilization of laboratory or simulation facilities^37^. With the experiment design, we can examine the changes in the desired response variable by applying changes to the input variables and control factors effective in experimenting. In light of the correct design of experiments, in addition to reducing costs, time, and number of experiments, more reliable results are obtained^38^. The two most common methods in RSM experiment design are designing experiments by CCD and BBD methods. The CCD or Central Composite Design method is one of the crucial and practical methods to reach the response surface (RSM) in many studies^39^. In this study, the CCD method was used for the relationship between responses and input variables, and finding the optimal value for output variables. The number of experiments in the CCD method compared to the BBD method is one of the reasons for selecting this method in this study, leading to obtaining more accurate and reliable results in conducting experiments and repeating some stages of experiments. Moreover, in the CCD method, each factor has 5 different levels (three points are inside the specified limits and two points are outside the specified limits). However, in the BBD method, each factor has 3 different levels and does not provide any point outside the specified limits for each factor. Thus, the beginning and end points of the range are less accurate than other points. Additionally, in the definition of the variables of this study, physical or conceptual limitations outside the desired range did not cause a problem in selecting the CCD method. In the CCD method, there are two numerical factors (X_1_, X_2_), which are respectively the weight percentage of nanoadsorbent in the range of 1 to 3% by weight and the solvent-to-fuel ratio in the range of 5–10 (in 5 levels), and a non-numerical factor (X_3_) indicating the type of nanoadsorbent (in 3 levels) were used. Table 2 shows the numerical and non-numerical independent factors used in this study.

**Table 2:** Numerical and non-numerical variables used in the design of the experiment.

Variables	Symbol	Coded variable levels				
		−α	−1	0	1	α+
Wt.% of nanostructure	X_1_	0.171573	1	3	5	5.82843
Solvent volume ratio	X_2_	3.96447	5	7.5	10	11.0355
Type of nanostructure	X_3_	MWCNT		MWCNT-Fe_2_O_3_	Silanated-Fe_2_O_3_	

Also, two variables R_1_ and R_2_, which are the values of remaining sulfur in the DAO phase in weight percentage and pitch yield, respectively, were used as CCD output.

## Discussion and conclusion

### Characterization of the synthesized nanostructures

#### XRD analysis

**Figure 3:**
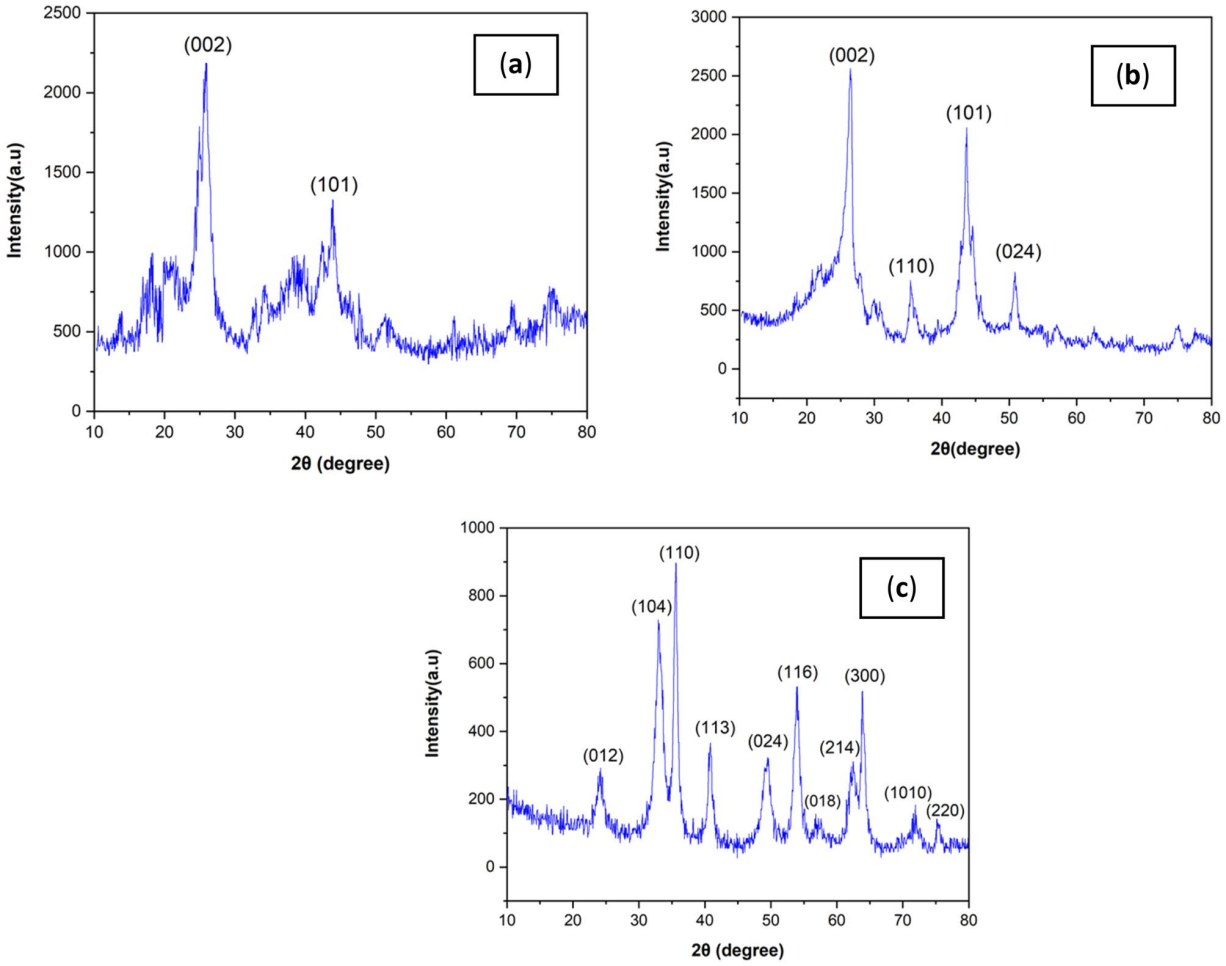
Diffraction pattern of nanoadsorbent: (**a**) MWCNT (**b**) MWCNT-Fe_2_O_3_ nanocomposite and (**c**) Silanated Fe_2_O_3_.

Figure 3a displays the X-ray diffraction pattern of the MWCNT sample. As shown in Fig. 3a, the primary diffraction peaks (θ2) at 26° and 43°, which are the same standard peaks related to carbon nanotube^40^, corresponding to (0 0 2) and (1 0 1) crystal planes, respectively, of sample and the standard card number (JCPDS no.41-1487). Figure 3b shows the X-ray diffraction pattern of the MWCNT-Fe_2_O_3_ nano-hybrid sample. As shown in Fig. 3b, the diffraction peaks (θ2) at 26.39° and 43.54° are the standard peaks, corresponding to the carbon nanotubes, respectively, with crystal planes (0 0 2) and (1 0 1) of the sample and the standard card (JCPDS no.41-1487). Also, the diffraction peaks (θ2) at 35.34° and 50.84°, respectively, with the crystal planes (1 1 0) and (0 2 4) of the standard sample of face center rhombohedral network of Fe_2_O_3_ correspond to the standard card number (JCPDS no. 89-0599). Figure 3c shows the X-ray diffraction pattern of the silanated-Fe_2_O_3_ nano-hybrid sample.

Based on the diffraction pattern, the crystal structure of the compound was examined. As shown in Fig. 3c, the diffraction peaks (θ2) at 24.12°, 33.11°, 35.61°, 40.82°, 49.41°, 54.00°, 57.50°, 62.38°, 63.94°, 71.82° and 75.40° with crystal planes (0 1 2), (1 0 4), (1 1 0), (1 1 3), (0 2 4), (1 1 6), (0 1 8), (2 1 4), (3 0 0), (1 0 10), and (2 2 0) of Fe_2_O_3_ standard sample correspond to standard card number (01-089-0688).

Based on software calculations, the mean crystallite size according to the two pairs of sharp peaks using Scherer's relation was found to be 35.11 nm for this sample. The synthetic compound is silanated with APTES, but since this compound does not show a characteristic peak in XRD, its functional groups were identified and confirmed by FTIR analysis. Also, the preservation and crystalline nature of the functionalized structure was confirmed by XRD analysis.

#### FTIR analysis

**Figure 4:**
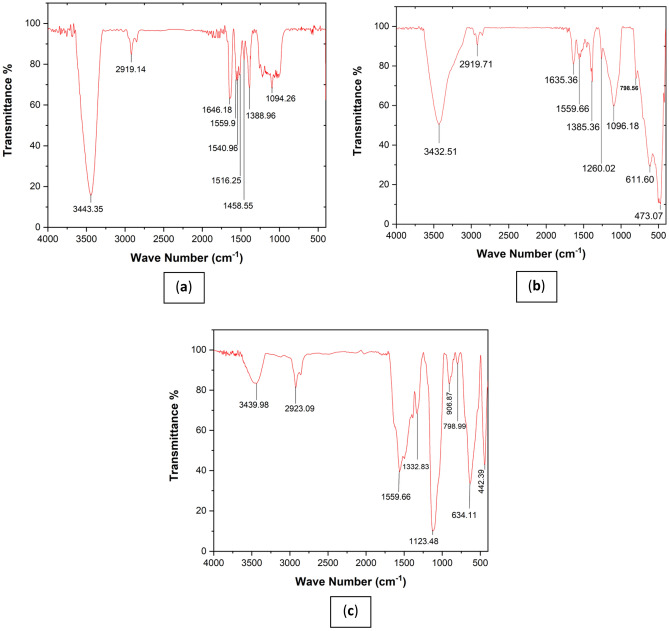
Fourier transform spectrum of nanostructure: (**a**) MWCNT, (**b**) MWCNT-Fe_2_O_3_, (**c**) Silanated-Fe_2_O_3_.

In the Fourier transform spectrum of MWCNT nano-adsorbent (Fig. 4a), the peak in the region above 3443 cm^−1^ confirms the presence of the OH hydroxyl group in the carbon nanotube structure, which is seen with very high intensity in the region above 3000 cm^−1^^41^. In the spectral analysis, a distinct peak appearing at approximately 2919 cm^−1^ exhibits a weak yet sharp intensity, signifying the stretching vibrations of the sp^3^ C–H group within the saturated system (comprising CH_2_ and CH_3_ groups). Additionally, it corresponds to the stretching vibrations of the sp^2^ C–H group inherent to the unsaturated alkene system present in the carbon nanotube structure, which predominantly manifests within this spectral range. Another noteworthy peak, found at around 1646 cm^−1^ with a moderately pronounced intensity, serves as an indicator for the stretching vibrations associated with the C=O group in the carbon nanotube structure^41,42^. Within the range of 1516–1559 cm^−1^, a peak of relatively moderate intensity reflects the stretching vibrations of the C=C alkene group within the carbon nanotube structure. Furthermore, a faint peak observed at 1458 cm^−1^ pertains to the scissor bending vibrations of the CH_2_ group within the carbon nanotube structure.

The spectrum also reveals a peak around 1388 cm^−1^, displaying a moderate intensity. This peak corresponds to the symmetric and asymmetric bending vibrations of the CH^3^ group within the compound structure. Finally, a prominent peak at 1094 cm^−1^ signifies the stretching vibrations of the C–O group in the carbon nanotube structure^43^. In the Fourier transform spectrum of the carbon nanotube sample, functionalized with Fe_2_O_3_ particles (Fig. 4b), the peak in the region above 3432 cm^−1^ confirms the presence of the OH hydroxyl group in the carbon nanotube structure, which is seen in the region above 3000 cm^−1^ due to its acidic nature and broadly due to the presence of hydrogen bond. As seen, the peak intensity in this region compared to the pure spectrum (peak in the region of 3443 cm^−1^) has appeared broadly, which confirms the hydrogen interaction between the OH group in the carbon nanotube structure and oxygen in the Fe_2_O_3_ structure. The peak observed at 2919 cm^−1^, displaying a sharp form and weak intensity, corresponds to the stretching vibrations of the sp^3^ C–H group within the saturated system (encompassing CH_2_ and CH_3_ groups). It also accounts for the stretching vibrations of the sp^2^ C–H group found in the unsaturated alkene system within the carbon nanotube structure. In the vicinity of 1635 cm^−1^, a peak with relatively weak intensity serves as an indicator for the stretching vibrations of the C=O group within the carbon nanotube structure. Notably, this peak's frequency and intensity differ from the pure spectrum (where the peak occurs at 1646 cm^−1^), confirming a dipole–dipole interaction between the oxygen of the carbonyl group in the carbon nanotube structure and the iron atom in the Fe_2_O_3_ structure. Another significant peak, situated at 1559 cm^−1^, is indicative of the stretching vibrations of the C = C alkene group within the carbon nanotube structure. Around 1385 cm^−1^, a peak of relatively moderate intensity relates to the symmetric and asymmetric bending vibrations of the CH_3_ group in the compound.

Furthermore, a strong-intensity peak at 1096 cm^−1^ is attributed to the stretching vibrations of the C–O group within the carbon nanotube structure. It's worth noting that the region below 1000 cm^−1^ in the IR spectrum is commonly referred to as the fingerprint region, where peaks are predominantly observed with weak intensity^44^.

Distinctive peaks include one at 611 cm^−1^, characterized by strong intensity, associated with the stretching vibrations of the Fe–O bond in the Fe_2_O_3_ structure, and another at 473 cm^−1^, also with strong intensity, which corresponds to the bending vibrations of the Fe–O group within the Fe_2_O_3_ structure^45^. In the Silanated-Fe_2_O_3_ Fourier transform spectrum (Fig. 4c), the peak above 3439 cm^−1^ confirms the presence of the NH_2_ amino group in the silanated compound, notably appearing broadly above 3000 cm^−1^ due to the presence of hydrogen bonds^46^. Additionally, a peak at 2923 cm^−1^, displaying relatively moderate intensity, is associated with the sp^3^ C–H stretching vibrations of the saturated system (comprising CH_2_ and CH_3_ groups) in the silanated compound structure. At 1559 cm^−1^, a peak with relatively strong intensity indicates the bending vibrations of the amino NH group within the structure. Around 1400 cm^−1^, a peak is attributed to scissor-like bending vibrations of the CH_2_ group in the silanated compound. The peak at 1332 cm^−1^, displaying moderate intensity, corresponds to the symmetric and asymmetric bending vibrations of the CH_3_ group in the compound.

Moreover, the peak at 1123 cm^−1^, characterized by strong intensity, is related to the stretching vibrations of the Si–O–C group in the silanated nanoabsorbent^32^. At 634 cm^−1^, a relatively strong-intensity peak is associated with the stretching vibrations of the Fe–O bond in the Fe_2_O_3_ structure^47^. Lastly, the peak at 442 cm^−1^ is indicative of the bending vibrations of the SiO_2_ group within the silanated structure. In light of these findings, the peaks at 2923 cm^−1^, 1559 cm^−1^ and 1123 cm^−1^ represent the formation of new groups when compared to pure Fe_2_O_3_, indicating the presence of silanated bonds on nano-Fe_2_O_3_ particles.

#### TEM analysis

**Figure 5:**
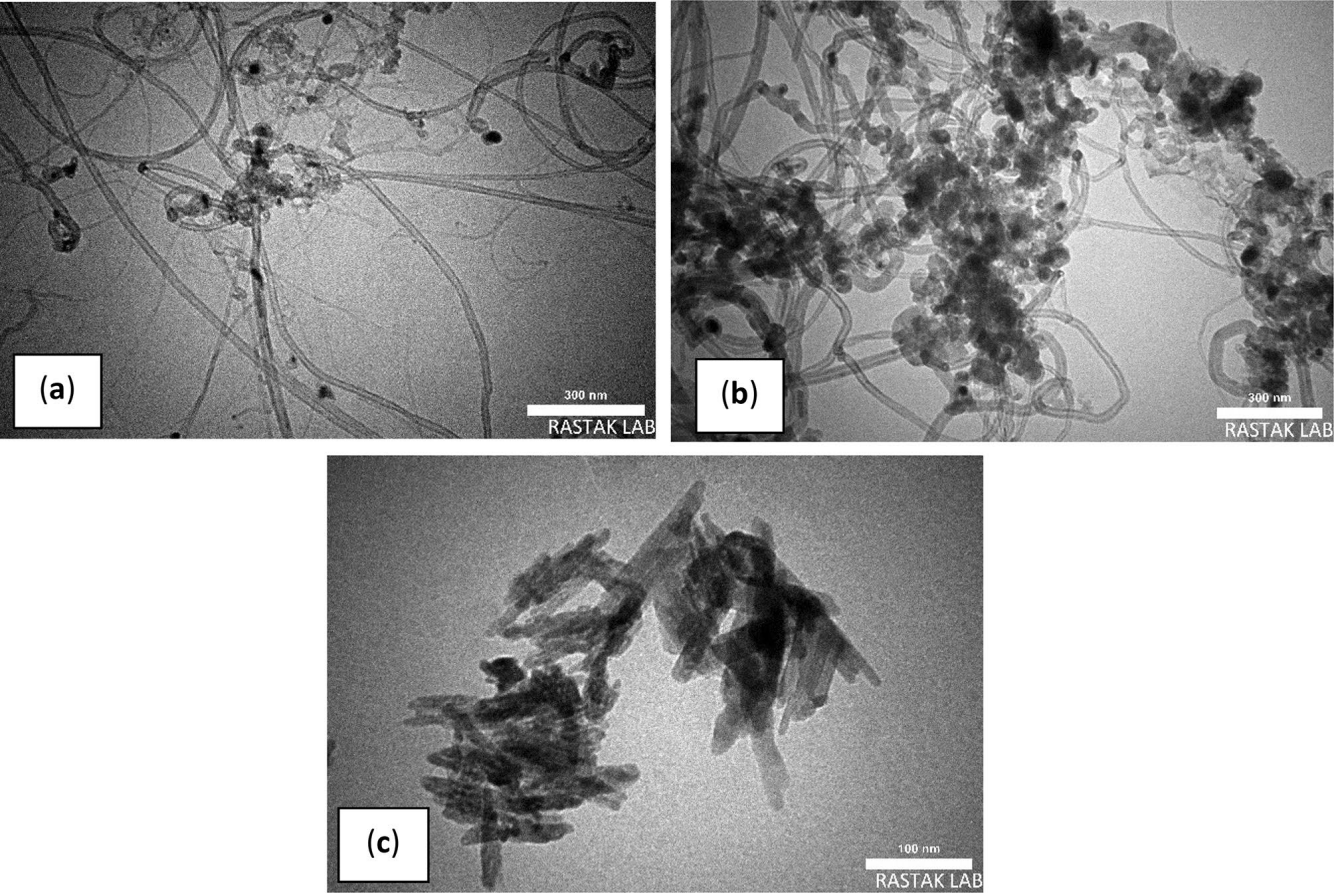
TEM images of synthesized nanoadsorbents of (**a**) MWCNT, (**b**) MWCNT-Fe_2_O_3_, (**c**) Silanated-Fe_2_O_3_.

Figure 5 shows the TEM images of synthesized MWCNT, MWCNT-Fe_2_O_3_, and Silanated-Fe_2_O_3_ nanoadsorbents. TEM images show the spaghetti-like structure of MWCNT and the average outer diameter of 10–12 nm (Fig. 5a).

The bold dots in the images of Fig. 5b and c show Fe_2_O_3_ and silanated particles, respectively, coated uniformly on the MWCNT and Fe_2_O_3_ surfaces. Additionally, the distribution of particles is uniform and the accumulation of particles is not seen. These images show the correct synthesis method of nanoadsorbents with appropriate efficiency in this study.

### Fit summary and ANOVA

The equation in terms of actual factors can be used to make predictions about the responses for given levels of each factor. In this study, the final equation in terms of actual factors is provided in Eqs. 2–7. Equations 2, 3 and 4 show the mathematical model for predicting the amount of residual sulfur in DAO, where the nano-absorbent is MWCNT, MWCNT-Fe_2_O_3_, and Silanated-Fe_2_O_3_, respectively. Equations 6, 5 and 7 show the mathematical model for predicting the value of Pitch Yield, where the nano-absorbent is MWCNT, MWCNT-Fe_2_O_3_, and Silanated-Fe_2_O_3_, respectively. The positive and negative signs of the coefficients in the obtained polynomial models indicate a positive or negative effect on the output variable. For example, in the model obtained for the amount of residual sulfur in DAO when Silanated-Fe_2_O_3_ nano-absorbent was used (Eq. 4), the sign of the coefficients of X_1_, X_1_^2^, and X_2_^2^ variables is positive, indicating the positive effect of these variables on the amount of residual sulfur in DAO. In contrast, the sign of X_2_ coefficients alone and the interaction effect of X_1_ and X_2_ variables (X_1_X_2_) are negative, indicating the negative effect of these variables on the amount of residual sulfur in DAO. An analysis of variance (ANOVA) was applied in order to investigate the adequacy of model. ANOVA results of these models was shown in Tables S1 and S2 for Response 1: sulfur content and Response 2: pitch yield, respectively.

As depicted in Table S1, the results of the ANOVA analysis demonstrate the model's significance, with a model *F*-value of 35.70. It’s worth noting that the likelihood of such a large *F*-value occurring by chance is a mere 0.01%. Furthermore, the presence of *P*-values less than 0.0500 indicates the significance of the model terms. Furthermore, Table S2 presents ANOVA results, revealing a significant model with an *F*-value of 34.60. The probability of an *F*-value of this magnitude arising from noise is only 0.01%. Additionally, the *P*-values below 0.0500 underscore the significance of the model terms. Based on the results presented in Table S1, the effect of variables X_1_, X_2_, and X_3_ alone is significant (*P*-value < 0.00001). Also, the interaction effect of X_1_X_2_ and X_1_X_3_ variables in determining the amount of residual sulfur in DAO is significant (*P*-value < 0.05). However, the interaction effect of X_2_X_3_ variables can be ignored. Moreover, the results presented in Table S2 indicate that the effects of X_1_, X_2_, and X_3_ variables alone and the interaction effects of X_1_X_3_ and X_2_X_3_ variables are significant in determining the value of Pitch Yield. However, the interaction effects of X_1_X_2_ variables can be ignored. In the obtained models for the amount of residual sulfur in DAO and also the amount of Pitch Yield by the CCD method, it was found that the type of nano-absorbent has the highest effect and the weight percentage of nano-absorbent has the lowest effect on the behavior of the output variables (X_3_ > X_2_ > X_1_).

**Figure 6:**
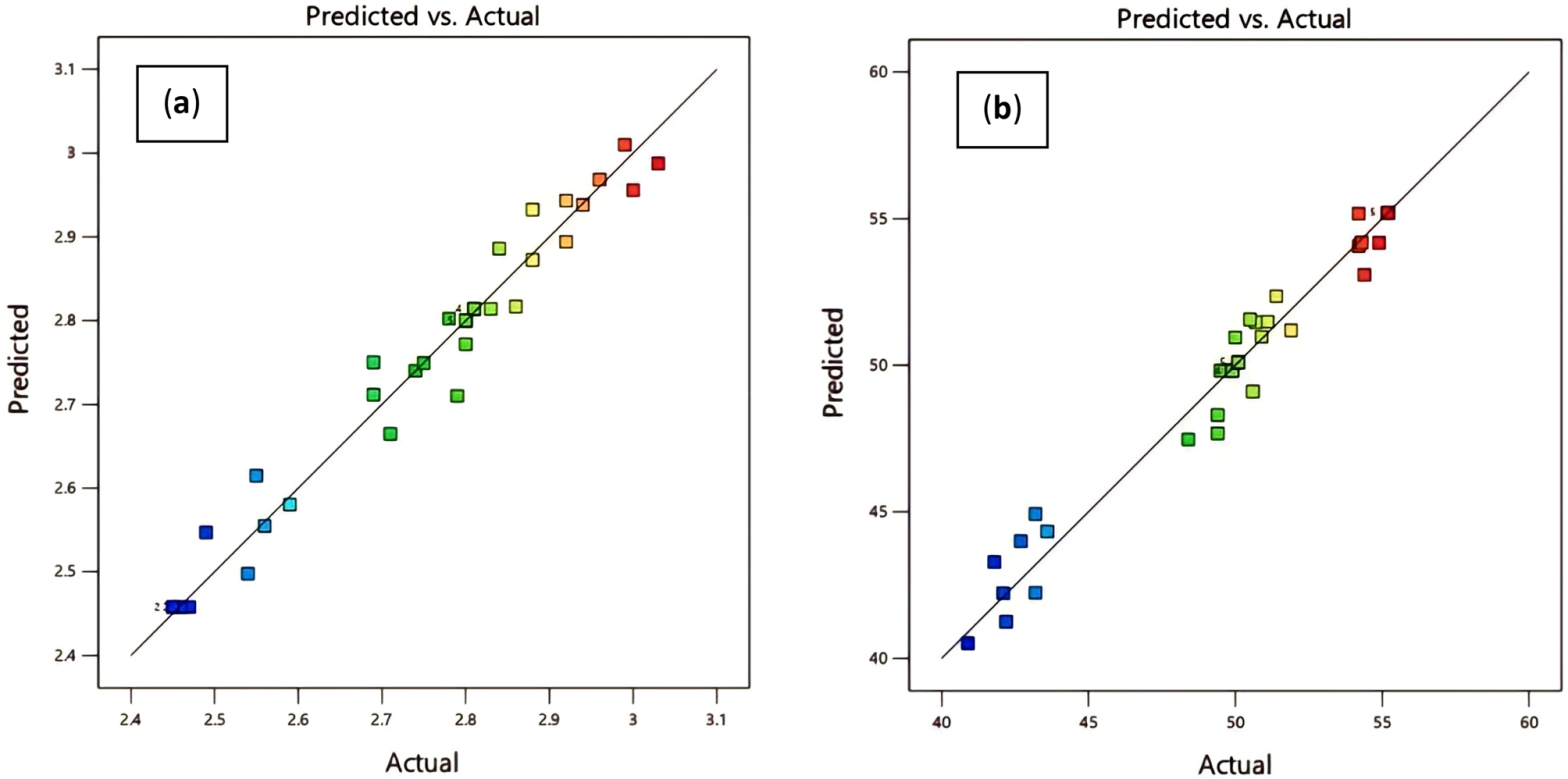
Models’ accuracy compares the experimental results to the mathematically statistically predicted value of the models: (**a**) for the sulfur content in DAO, (**b**) pitch yield.

The Predicted R^2^, standing at 0.7559, aligns reasonably well with the adjusted R^2^ of 0.9376, reinforcing the model's predictability and acceptability. As shown in Fig. 6, the models accuracy compares the experimental results to the mathematically statistically predicted value of the models for the sulfur content in DAO and pitch yield.

2 $${Y}_{1}=3.50838-0.065517{X}_{1}-0.118878{X}_{2}-0.001500{X}_{1}{X}_{2}+0.007000{X}_{1}^{2}+0.006480{X}_{2}^{2}$$

3 $${Y}_{1}=3.23786-0.023321{X}_{1}-0.069678{X}_{2}-0.007500{X}_{1}{X}_{2}+0.009688{X}_{1}^{2}+0.004200{X}_{2}^{2}$$

4 $${Y}_{1}=3.50409+0.031581{X}_{1}-0.282986{X}_{2}-0.009500{X}_{1}{X}_{2}+0.008062{X}_{1}^{2}+0.019960{X}_{2}^{2}$$

5 $${Y}_{2}=17.35981+4.72943{X}_{1}+5.25625{X}_{2}-0.030000{X}_{1}{X}_{2}-0.465000{X}_{1}^{2}-0.289600{X}_{2}^{2}$$

6 $${Y}_{2}=20.48149+3.01095{X}_{1}+5.15225{X}_{2}+0.095000{X}_{1}{X}_{2}-0.493750{X}_{1}^{2}-0.280000{X}_{2}^{2}$$

7 $${Y}_{2}=42.53892-1.13188{X}_{1}+3.73464{X}_{2}+0.180000{X}_{1}{X}_{2}-0.065625{X}_{1}^{2}-0.274000{X}_{2}^{2}$$

### DAO analysis

As stated in Section 4.2, to examine the effective parameters in the deasphalting of fuel oil to achieve a lower amount of sulfur in fuel oil, different tests based on the three variables of nanostructure percentage added to the fuel (X_1_), solvent-to-fuel ratio (X_2_), and type of nanoadsorbent (X_3_) were designed and implemented using CCD method. The DAO (R_1_) and yield pitch (R_2_) were measured according to the experimental design in 39 steps, as presented in Table 3. The variable range of X_1_ was from 1 to 3% by weight, the variable range of X_2_ was from 5 to 10, and the variable type of X_3_ was considered as MWCNT, MWCNT-Fe_2_O_3_, and Silanated Fe_2_O_3_, respectively.

**Table 3:** The design of the experiment used in this study with the CCD method.

Run	Factor 1	Factor 2	Factor 3	Response 1	Response 2
	A: nano conc. (wt.%)	B: solvent to oil ratio	C: nano type	Sulfur content (wt.%)	Pitch yield (wt.%)
1	3	7.5	Silane-Fe_2_O_3_	2.45	55.2
2	0.171573	7.5	MWCNT	2.96	42.2
3	5.82843	7.5	MWCNT	2.8	50
4	3	7.5	MWCNT-Fe_2_O_3_	2.8	50.1
5	1	10	MWCNT-Fe_2_O_3_	2.88	48.4
6	5	10	Silane-Fe_2_O_3_	2.56	54.3
7	3	7.5	MWCNT-Fe_2_O_3_	2.8	50.1
8	5.82843	7.5	MWCNT-Fe_2_O_3_	2.86	49.4
9	5	5	MWCNT-Fe_2_O_3_	2.88	43.6
10	3	7.5	MWCNT	2.83	49.5
11	3	7.5	MWCNT-Fe_2_O_3_	2.8	50.1
12	3	11.0355	MWCNT	2.78	50.6
13	3	11.0355	Silane-Fe_2_O_3_	2.71	51.4
14	1	10	MWCNT	2.92	43.2
15	1	5	MWCNT-Fe_2_O_3_	2.92	43.2
16	3	7.5	MWCNT	2.81	49.9
17	1	5	MWCNT	2.99	40.9
18	3	11.0355	MWCNT-Fe_2_O_3_	2.75	50.9
19	3	7.5	MWCNT-Fe_2_O_3_	2.8	50.1
20	3	7.5	Silane-Fe_2_O_3_	2.45	55.2
21	3	3.96447	MWCNT-Fe_2_O_3_	3	42.1
22	5	5	MWCNT	2.84	49.4
23	3	7.5	MWCNT	2.81	49.9
24	5	10	MWCNT	2.74	51.1
25	3	7.5	Silane-Fe_2_O_3_	2.46	55.2
26	5	5	Silane-Fe_2_O_3_	2.79	50.5
27	3	7.5	MWCNT	2.81	49.9
28	5.82843	7.5	Silane-Fe_2_O_3_	2.49	54.9
29	3	3.96447	MWCNT	3.03	41.8
30	3	3.96447	Silane-Fe_2_O_3_	2.69	51.9
31	3	7.5	MWCNT-Fe_2_O_3_	2.8	50.1
32	0.171573	7.5	MWCNT-Fe_2_O_3_	2.94	42.7
33	3	7.5	Silane-Fe_2_O_3_	2.47	55.2
34	1	5	Silane-Fe_2_O_3_	2.59	54.2
35	3	7.5	Silane-Fe_2_O_3_	2.46	55.2
36	1	10	Silane-Fe_2_O_3_	2.55	54.4
37	0.171573	7.5	Silane-Fe_2_O_3_	2.54	54.2
38	5	10	MWCNT-Fe_2_O_3_	2.69	50.7
39	3	7.5	MWCNT	2.81	49.9

**Figure 7:**
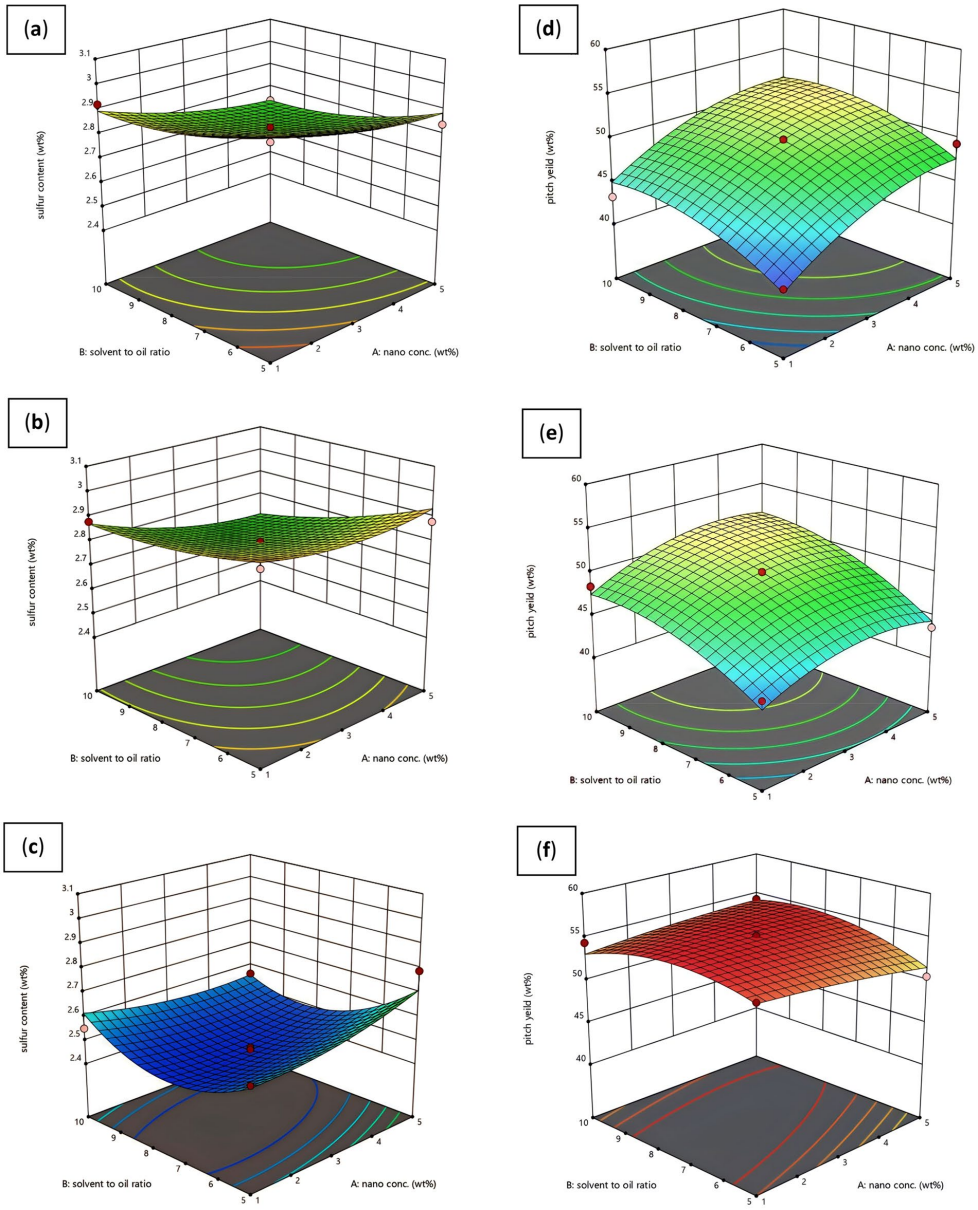
Three-dimensional graph of surface response: (**a**) R_1_ output and use of MWCNT nanostructure (**b**) R_1_ output and use of MWCNT-Fe_2_O_3_ nanostructure (**c**) R_1_ output and use of silanated-Fe_2_O_3_ nanostructure (**d**) R_2_ output and use of nanostructure MWCNT structure (**e**) R_2_ output and use of MWCNT-Fe_2_O_3_ nanostructure (**f**) R_2_ output and use of Silanated-Fe_2_O_3_ nanostructure.

To understand the effect of the parameters on the output variables well, 3D graphs of the surface response were used, as shown in Fig. 7. Graphs **a**, **b** and **c** in Fig. 7 show the behavior of the weight percentage of sulfur remaining in DAO against the effect of the solvent-to-fuel ratio and the weight percentage of added nanostructures in three types of MWCNT, MWCNT-Fe_2_O_3_, and Silanated-Fe_2_O_3_, respectively. In the graph of Fig. 7a, based on the obtained surface response, when the added nanoadsorbent weight and the solvent-to-fuel ratio are higher, the amount of final sulfur in DAO will be lower.

The reason for a reduction in sulfur percentage in DAO with the increase in the solvent-to-fuel ratio is related to the strength of solvent solubility. By increasing the solvent-to-fuel ratio, the value of non-asphaltenic substances, including resins, is dissolved in the normal heptane solvent in a larger amount. In this state, resinous substances cannot act as surface agents for asphaltene particles, leading to the accumulation and deposition of asphaltene particles^48^. Due to an increase in the deposition of asphaltene particles, larger amounts of heavy sulfur compounds are removed from the fuel. It can be seen based on the results of DAO phase sulfur analysis.

The behavior of the input variables in the graph of Fig. 7b and in the state of using the MWCNT-Fe_2_O_3_ nanostructure is very similar to the state of using the pure MWCNT nanostructure. For MWCNT nanoparticles, the π-π bond between the electron superstructure of the aromatic rings of carbon nanotubes and asphaltene leads to the adsorption of asphaltene on the carbon nanotubes^47^. It finally becomes larger and heavier over time and causes more deposition of asphaltene particles in the fuel, leading to an increase in the removal percentage of asphaltene. It causes more removal of heavy sulfur compounds from the fuel and increases the fuel quality.

**Figure 8:**
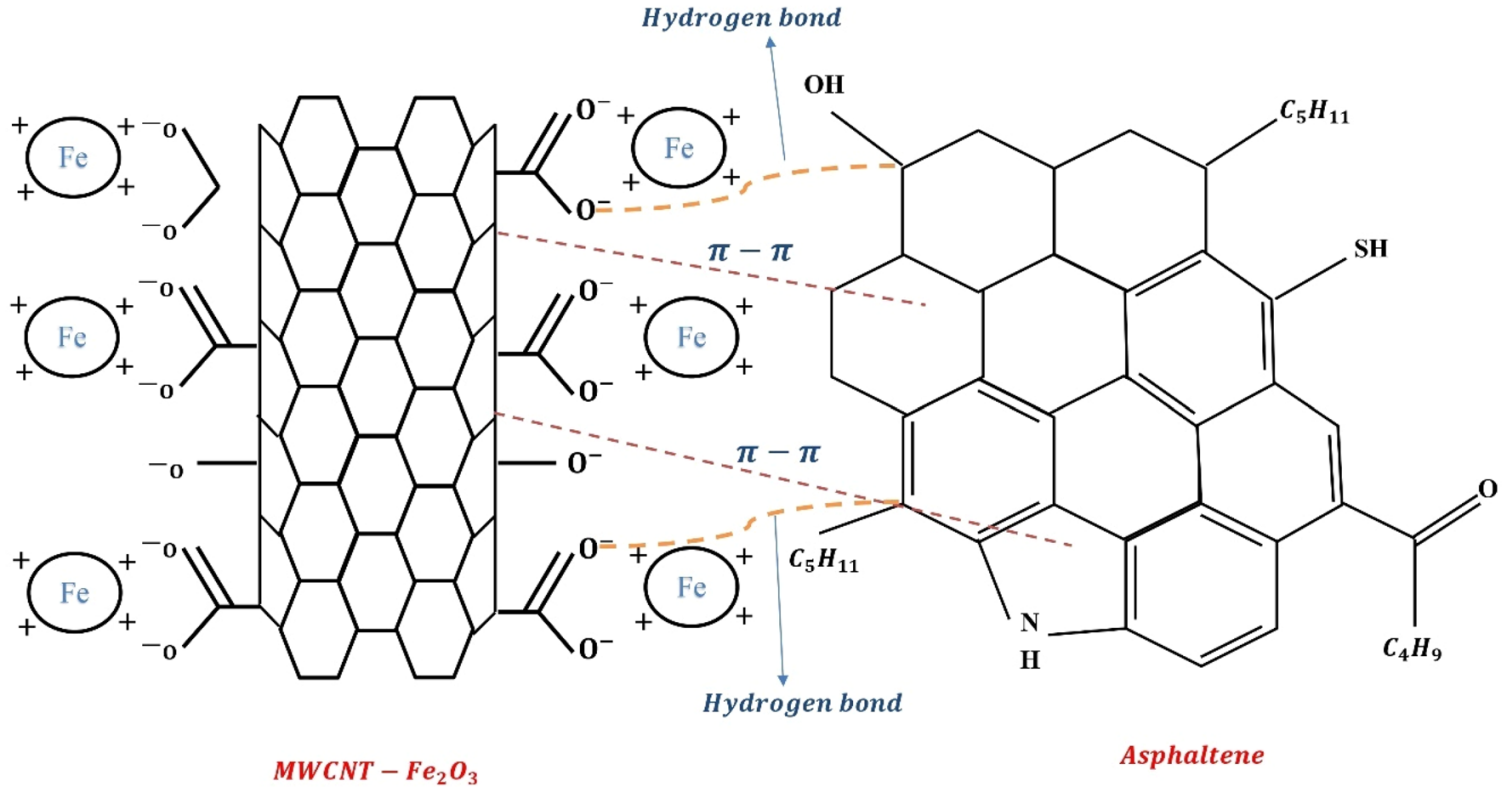
Mechanism of hydrogen bonding versus π–π bonding.

The presence of Fe element in MWCNT-Fe_2_O_3_ nanohybrid leads to the formation of a strong molecular interaction and bond such as hydrogen bond, acid and base interaction, and electrostatic force interaction between nanoadsorbent and asphaltene molecules. By comparing the two three-dimensional Graphs **a** and **b** in Fig. 7, it is revealed that when MWCNT-Fe_2_O_3_ nanohybrid is used in the fuel (at a high amount of solvent), sulfur reduction is greater than when the pure MWCNT nanostructure has been used. Its reason can be attributed to the stronger hydrogen bond in the bond between asphaltene molecules with MWCNT-Fe_2_O_3_ compared to the π–π bond of pure carbon nanotubes with asphaltene molecules (Fig. 8). The hydrogen bond is formed between the hydrogen atom of the functional group on MWCNT-Fe_2_O_3_ (e.g., –OH, –COOH) and the oxygen atom of the asphaltene molecule. This provides a strong attraction between the two entities, enhancing the adsorption capacity of MWCNT-Fe_2_O_3_ for asphaltene. The π–π bond in pure MWCNT, on the other hand, is weaker compared to the hydrogen bond in MWCNT-Fe_2_O_3_. The π–π bond arises from the overlapping of π electrons in the carbon nanotube structure and can interact with the aromatic rings in the asphaltene molecules. While this bond can contribute to asphaltene removal, it is generally not as strong as the hydrogen bond.

According to Graph **c** in Fig. 7, with the increase in the weight percentage of Silanated-Fe_2_O_3_ nanoadsorbent, the percentage of sulfur remaining in the fuel first decreases and then increases. To examine this difference, the API test was performed at the point where the lowest percentage of sulfur was obtained compared to the point where the highest percentage of silanated nanoadsorbent and the highest solvent-to-fuel ratio were obtained. It was revealed that the API in the middle point of the surface response graph has a lower value compared to the endpoint of the graph (the highest value of nanoadsorbent percentage and solvent-to-fuel ratio).

Although there is the lowest amount of sulfur in the fuel, a lighter fuel has been obtained in the numbers at the end point of the graph, indicating the higher adsorption of asphaltene particles by the Silanated-Fe_2_O_3_ nanostructure. The surface charge created by Silanated particles makes asphaltene particles more stable in fuel. In the middle point of the graph, the asphaltene particles adsorbed by Silanated-Fe_2_O_3_ particles are attracted to each other and the increase in molecular weight due to the gravitational force causes more deposition, leading to the removal of a larger amount of asphaltene from the fuel. Hence, sulfur decreases significantly in fuel which is very favorable.

Graphs **d**, **e**, and **f** in Fig. 7 show the behavior of yield pitch against the effect of solvent-to-fuel ratio and weight percentage of added nanostructure in three types of MWCNT, MWCNT-Fe_2_O_3_, and Silanated-Fe_2_O_3_, respectively. By pairwise comparing graphs **a** and **d**, **b** and **e**, and **c** and **f** in Fig. 7, it was found that the point of the graph where the lowest amount of sulfur in the fuel was obtained is the same as the state where the highest yield pitch was obtained.

It means that more asphaltene deposition in the fuel leads to a decrease in the percentage of sulfur in the fuel, indicating the removal of heavy sulfur compounds in fuel oil.

#### Optimization

To find the optimal conditions of variables X_1_, X_2_, and X_3_ to minimize the variable R_1_ (or maximize the variable R_2_), the optimization was performed using a numerical optimization module. It uses models to find the specific point for the best conditions bringing the desired function to the minimum (for R_1_) or maximum (for R_2_). Based on the optimization performed, to reach the minimum amount of sulfur in DAO (R_1_) or to maximize the pitch yield (R_2_), the optimized values are presented in Table 4.

**Table 4:** Optimized values for three variables X_1_, X_2_, and X_3_ to minimize the R_1_ variable or maximize the R_2_ variable.

Nano conc (X_1_)	Solvent to oil ratio (X_2_)	Nano type (X_3_)	Sulfur content	Pitch yield	Desirability
2.581	7.704	Silanated-Fe_2_O_3_	2.455	55.269	0.991

To validate the model optimal conditions, a validation test was performed using optimal values and the percentage of sulfur remaining in DAO and pitch yield was measured, as shown in Table 5.

**Table 5:** Optimized values for the variables used in this study and comparing the experimental values obtained in these conditions with the values calculated by the model.

Sulfur in DAO (wt.%)		Pitch yield	
Predictive	Experimental	Predictive	Experimental
2.455	2.41	55.269	55.18

Since the experimental values and the data predicted by the model are close to each other, the validity of the model for predicting the weight percentage of remaining sulfur in DAO and Pitch yield is well proven.

#### The effect of temperature

**Figure 9:**
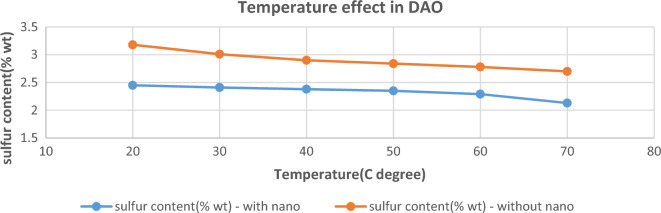
The effect of temperature on the remaining sulfur in DAO with and without the presence of Silanated-Fe_2_O_3_ nanoadsorbent in optimal conditions.

To examine the effect of increasing temperature on the quality of DAO, the deasphalting process was performed in the temperature range of 20°–70 °C. Figure 9 shows the percentage of remaining sulfur in DAO with the presence and absence of nanoadsorbent. In the experiments, the parameters effective in deasphalting, such as the type of nanoadsorbent, the weight percentage of nanoadsorbent, and the solvent-to-fuel ratio were kept constant and their values were chosen based on the most optimal state in the design of the experiment (Silanated-Fe_2_O_3_ nanoadsorbent with 2.579 wt.% and 7.704 solvent-to-fuel ratio). The results obtained in the graph of Fig. 9 showed that in both states of the presence and absence of nanoadsorbent, the remaining sulfur weight percentage in DAO decreases with increasing temperature, leading to higher DAO quality. In the case of not using nanoadsorbent, the weight percentage of remaining sulfur in DAO reached 2.7 at 70 °C, indicating a decrease of 22.85% compared to the primary fuel.

In this case, increasing the temperature causes a difference in the solubility parameters and molar volume between the solvent and the fuel, which creates a broader region in the solvent-asphaltene-DAO ternary system. Solubility parameters represent the cohesive energy density of a substance and can be used to determine its compatibility or solubility with other substances. Molar volume, on the other hand, is a measure of the amount of space occupied by one mole of a substance. As the temperature increases in the ternary system, the solubility parameters of the components may change. Solvents with higher solubility parameters tend to have better solvating power for asphaltenes and DAO, which are complex organic compounds derived from crude oil. This means that at higher temperatures, the solvents can dissolve a larger amount of asphaltenes and DAO^49,50^. In the case of using Silanated-Fe_2_O_3_ nano-adsorbent, the weight percentage of remaining sulfur in DAO reached 2.13 at 70 °C, indicating a 39.14% decrease compared to the primary fuel. This case indicates a synergistic effect between nanoadsorbent and temperature, which improves the quality of DAO. Its reason may be the adsorption of more amounts of undissolved asphaltene in the solvent on the surface of the nanostructure due to the increase in thermal motion. As the thermal motion of molecules increases, their random movement and collisions with the nanostructure surface become more frequent. This increased collision probability leads to a higher chance of asphaltene molecules coming into contact with the surface and being adsorbed. Furthermore, increasing diffusion thermal motion provides energy to asphaltene molecules, enabling them to overcome energy barriers and diffuse across the surface. This enhanced diffusion allows for greater spreading of the asphaltene molecules on the nanostructure surface, increasing the overall adsorption^18^.

As temperature increases, the density of DAO generally decreases. This is because the molecules in DAO expand and occupy a larger volume as they gain thermal energy. The expansion leads to a decrease in the overall density of the oil. Lower density means that the DAO is less compact, and the individual molecules are spaced further apart. Increasing temperature also decreases the viscosity of DAO. Viscosity refers to the resistance of a fluid to flow, and it is determined by the intermolecular forces between the molecules of the fluid. At higher temperatures, these intermolecular forces weaken, and the fluid molecules can move more freely, reducing the viscosity.

### Pitch analysis

**Figure 10:**
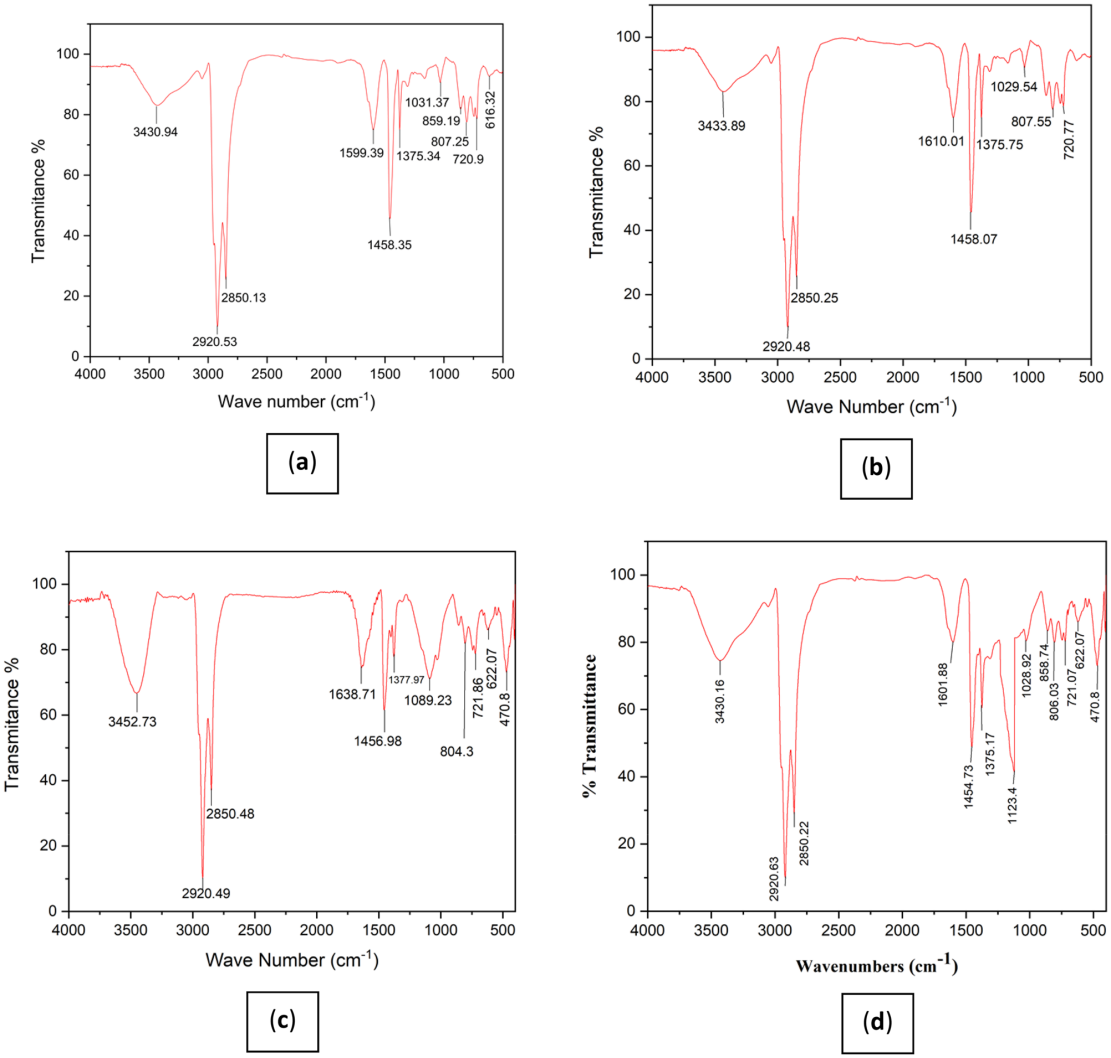
Fourier transform spectrum: (**a**) pure asphaltene, (**b**) asphaltene adsorbed by MWCNT, (**c**) asphaltene adsorbed by MWCNT-Fe_2_O_3_, (**d**) asphaltene adsorbed by Silanated-Fe_2_O_3_.

After performing the experiments, FTIR and FESEM analysis were performed to examine the mechanism and behavior of asphaltene adsorption on the surface of nanoadsorbents. The obtained results are discussed in this section. For a more accurate comparison, pure asphaltene without the use of nanoadsorbent was also extracted from fuel oil by the IP-143 standard method. Figure 10 shows the Fourier transform infrared spectrometer analysis of pure asphaltene and asphaltene adsorbed on nanoadsorbent surfaces to examine the growth mechanism and adsorption mechanism. Part **a** in Fig. 10 shows the Fourier transform of the pure asphaltene sample.

The peak in the region above 3430 cm^−1^ confirms the presence of the OH hydroxyl group in the asphaltene structure, which is seen broader in the region above 3000 cm^−1^ due to the presence of hydrogen bonds. In the spectral analysis, a peak spanning from 2920 to 12,850 cm^−1^, characterized by strong intensity, corresponds to the stretching vibrations of the sp3 C–H group within the saturated system, encompassing CH_2_ and CH_3_ groups. It also accounts for the stretching vibrations of the sp^2^ C–H group within the unsaturated alkene and aromatic system in the asphaltene structure, which predominantly manifests in this spectral region. At around 1599 cm^−1^, a peak of relatively moderate intensity serves as an indicator, signifying the stretching vibrations of the C=C alkene and aromatic group in the asphaltene structure.

A moderate-intensity peak at 1458 cm^−1^ relates to the scissor-like bending vibrations of the CH_2_ group in the asphaltene structure. Around 1375 cm^−1^, another peak with moderate intensity is associated with the symmetric and asymmetric bending vibrations of the CH_3_ group within the compound. Additionally, a relatively weak-intensity peak at 1031 cm^−1^ is related to the stretching vibrations of the C–O group and the stretching vibrations of the S=O sulfoxide group in the asphaltene structure^51^.

In the spectral range spanning 720–859 cm^−1^, peaks are related to the C–H bending vibrations of the aromatic and alkene systems, appearing in this region with weak intensity. Figure 10b displays the Fourier transform of the asphaltene sample adsorbed on the MWCNT surface, revealing a peak above 3433 cm^−1^, which confirms the presence of the OH hydroxyl group within the asphaltene and carbon nanotube structures. This peak is broadly evident in the region above 3000 cm^−1^, attributable to the presence of hydrogen bonds. In the spectral region spanning 2850–2920 cm^−1^, a strong-intensity peak corresponds to the sp^3^ C–H stretching vibrations of the saturated system (CH_2_ and CH_3_) within the carbon nanotube compound, which predominantly manifests in this spectral region. At approximately 1610 cm^−1^, a peak of relatively moderate intensity indicates the stretching vibrations of the C=O group within the carbon nanotube structure. Another peak, located at 1458 cm^−1^, is associated with the scissor-like bending vibrations of the CH_2_ group in the carbon nanotube structure.

Around 1375 cm^−1^, a peak of moderate intensity signifies the symmetric and asymmetric bending vibrations of the CH_3_ group within the compound. Moreover, a peak at 1029 cm^−1^, displaying relatively weak intensity, is related to the stretching vibrations of the C–O group within both the asphaltene and carbon nanotube compounds.

Figure 10c illustrates the Fourier transform of the asphaltene sample adsorbed on the MWCNT-Fe_2_O_3_ surface. The peak in the region above 3452 cm^−1^ confirms the presence of the OH hydroxyl group in the structure of asphaltene and carbon nanotubes, seen in the region above 3000 cm^−1^ due to its acidic characteristic and broadly due to the presence of hydrogen bonds. As seen, the frequency of this region has increased compared to the pure spectrum (peak in the region of 3433 cm^−1^), confirming the hydrogen interaction between the OH group in the carbon nanotube structure and oxygen in the Fe_2_O_3_ structure. The peak in the region of 2850–2920 cm^−1^ with strong intensity is related to the sp^3^ C–H stretching vibrations of the saturated system (CH_2_ and CH_3_) in the structure of the carbon nanotube compound. The peak in the region of 1638 cm^−1^ with relatively moderate intensity is an indicator related to the stretching vibrations of the C=O group of the carbon nanotube structure.

As seen, the frequency and intensity of the peak of the carbonyl group in this region have slightly increased compared to the pure spectrum (peak in the region of 11,610 cm^−1^), confirming the dipole–dipole interaction between the oxygen of the carbonyl group in the carbon nanotube structure and the iron atom in the Fe_2_O_3_ structure.

The peak in the approximate region of 1456 cm^−1^ is related to the scissor-like bending vibrations of the CH_2_ group in the carbon nanotube structure. The peak in the region of 1377 cm^−1^ with relatively moderate intensity is related to the symmetric and asymmetric bending vibrations of the CH_3_ group in the compound. Also, the peak in the region of 1089 cm^−1^ with strong intensity is related to the stretching vibrations of the C–O group in the structure of asphaltene and carbon nanotube compounds (by adding Fe_2_O_3_ to the compound, the intensity and frequency of the peak in this region increased significantly). The peak at 622 cm^−1^ with relatively weak intensity is related to the stretching vibrations of the Fe–O bond in the Fe_2_O_3_ structure and the peak at 470 cm^−1^ with moderate intensity is related to the bending vibrations of the Fe–O group in the Fe_2_O_3_ structure.

Comparing spectrum **c** in Fig. 10 with spectrum **b** in Fig. 10 shows that MWCNT-Fe_2_O_3_ nanoadsorbent has a higher efficiency than MWCNT to control the formation of asphaltene and the size growth of asphaltene particles, indicating the higher adsorption power of asphaltene particles on the surface of MWCNT-Fe_2_O_3_ nanoadsorbent compared to MWCNT. Part **d** in Fig. 10 shows the Fourier transform of the asphaltene sample adsorbed on the Silanated-Fe_2_O_3_ surface.

Evident in the spectral analysis is a peak situated above 3430 cm^−1^, serving as confirmation of the presence of the OH hydroxyl group within the asphaltene structure. This peak is notably broad in the region above 3000 cm^−1^, attributable to the presence of hydrogen bonds. The region spanning 2850–2920 cm^−1^ features a robust-intensity peak associated with the sp^3^ C–H stretching vibrations of the saturated system, encompassing CH_2_ and CH_3_ groups, both in the asphaltene structure and the silanated compound. Additionally, it corresponds to the sp^2^ C–H stretching vibrations of the alkene and aromatic saturated system within the asphaltene structure, predominantly manifesting in this spectral range. At approximately 1601 cm^−1^, a peak of relatively moderate intensity signifies the stretching vibrations of the C=C alkene and aromatic group within the asphaltene structure. The region around 1454 cm^−^ contains a moderate-intensity peak related to the scissor-like bending vibrations of the CH_2_ group in both the asphaltene structure and the silanated compound. Furthermore, around 1375 cm^−1^, a peak with moderate intensity corresponds to the symmetric and asymmetric bending vibrations of the CH_3_ group within the structure. A very strong-intensity peak at 1123 cm^−1^ is related to the stretching vibrations of the Si–O group within the silanated structure. In the region of 1028 cm^−1^, a weak-intensity peak is associated with the stretching vibrations of the C–O group in the asphaltene structure. Within the spectral range spanning 721–854 cm^−1^, peaks relate to the aromatic C–H and alkene bending vibrations within the asphaltene structure, appearing with weak intensity. At 622 cm^−1^, a peak of relatively weak intensity signifies the stretching vibrations of the Fe–O bond in the Fe_2_O_3_ structure. A moderate-intensity peak at 470 cm^−1^ corresponds to the bending vibrations of the SiO_2_ group in the silanated structure.

**Figure 11:**
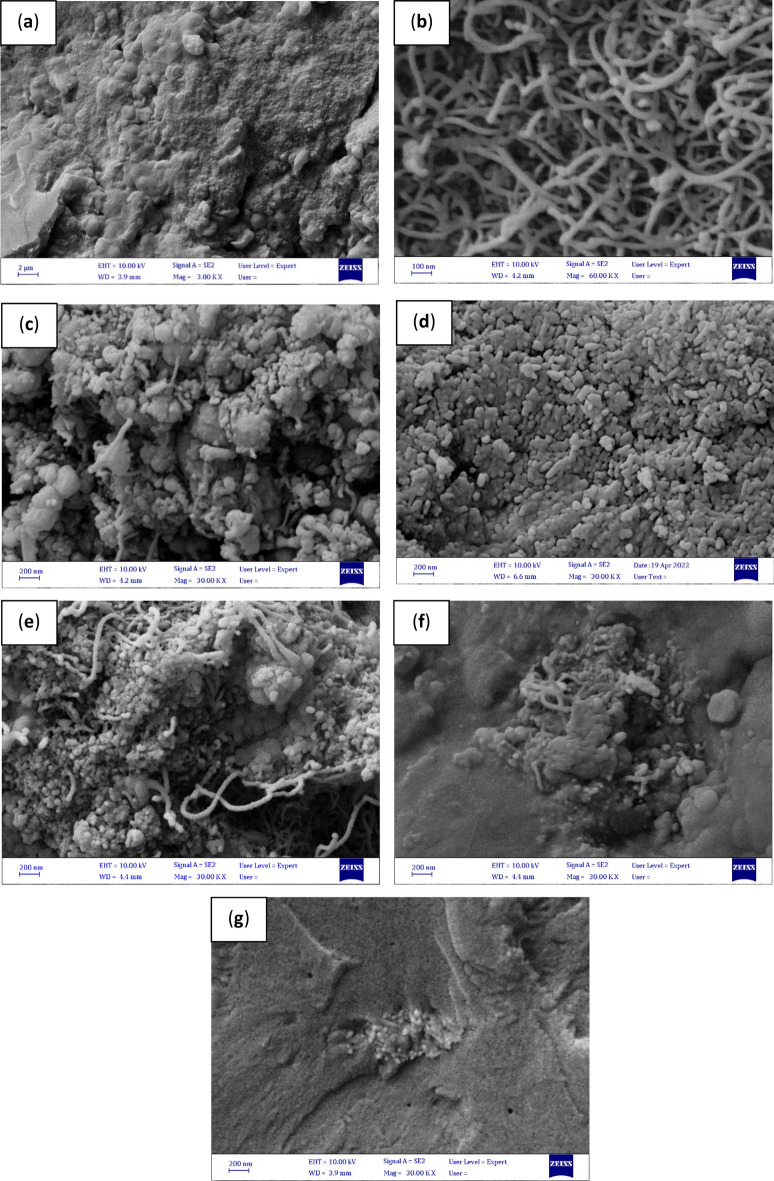
FESEM images: (**a**) pure asphaltene (**b**) pure MWCNT (**c**) pure MWCNT-Fe_2_O_3_ (**d**) pure Silanated-Fe_2_O_3_ and (**e**), (**f**), (**g**) asphaltenes adsorbed on MWCNT, MWCNT-Fe_2_O_3_, and Silanated-Fe_2_O_3_ surfaces, respectively.

The size and morphology of pure asphaltene particles and nanoadsorbents before and after asphaltene adsorption underwent analysis using the FESEM technique, as depicted in Fig. 11. In Fig. 11a, the FESEM image of pure asphaltene particles reveals two distinct structural patterns, characterized by both smooth and uneven surfaces with deep grooves and fractures, indicating highly favorable conditions for the deposition of agglomerate particles. Figure 11b shows the image of MWCNT particles. Its spaghetti-like structure, which is the result of growth on Co-Mo/MgO, is well shown. Their average size is 12–10 nm. Also, the uniform distribution of particles can be seen well, indicating the appropriate method for its synthesis. Figure 11c shows the image of Fe_2_O_3_ particles engaged well with carbon nanotube particles and uniformly distributed on the surfaces. The average Fe_2_O_3_ particles placed on multi-walled carbon nanotubes are in the range of 35–40 nm. Figure 11d shows the image of the Silanated-Fe_2_O_3_ hybrid nanoadsorbent, showing the uniform distribution of silanated particles on iron nano-oxide. The largest particle size distribution in this case is in the range of 20–40 nm. Comparing the FESEM image of pure asphaltene and pure nanostructures with the FESEM images of asphaltene particles adsorbed on the asphaltene surface (Fig. 11e–g) shows that the morphology of the surfaces has turned into an irregular pattern.

It confirms the strong bonds between nanoadsorbents and asphaltene. Also, the created irregular patterns are related to the adsorption of asphaltenes on the surfaces of nanoadsorbents, leading to a weaker interaction of asphaltenes with adjunct particles.

This issue confirms the deposition of more asphaltene particles adsorbed on the nanoadsorbent surfaces in the fuel, leading to more deasphalting and a greater reduction of sulfur from the fuel.

Table 6 shows the comparison between this study and similar studies based on the solvent deasphalting method. Based on the findings, it is evident that this research demonstrates a notable proficiency in eliminating sulfur from heavy fuel oil through the solvent deasphalting process. Furthermore, economic aspects such as the quantity of solvent to oil ratio and the weight percentage of nanoabsorbent were taken into account, making the study outperform other similar investigations.

**Table 6:** Comparing the findings of this research to those of related studies.

Feed	Methodology	Sulfur removal (%)	References
Colombian extra-heavy crude oil	SDA + Adding nanoadsorbents (Sio_2_)	23	^49^
Heavy vacuum fraction	SDA + Adding zeolite adsorbent and vanadium xerogel	31	^30^
Heavy oil	SDA + Hydrotreating	35 (At HDAO-4 sample)	^52^
Iraqi heavy crude oil	SDA + Adding nano silica adsorbents	51.17 (At 7 wt.% nano silica and SOR = 12	^29^
Heavy Mazut fuel oil	SDA + Adding nanoadsorbents (MWCNT, MWCNT-Fe_2_O_3_, and Silanated-Fe_2_O_3_)	40 (At 2.581 wt.% Silanated-Fe_2_O_3_ and SOR = 7.704	This work

## Conclusion

Solvent deasphalting process is one of the methods that can be used to reduce the amount of sulfur in petroleum compounds. In this study, the process of solvent deasphalting from heavy Mazut fuel oil, by adding three types of nanoadsorbents (MWCNT, MWCNT-Fe_2_O_3_, and Silanated-Fe_2_O_3_), demonstrated that a fuel with a lower weight percentage of sulfur was obtained compared to the traditional solvent deasphalting process. The use of CCD/RSM method in Design Expert software for this work shows the influence of effective parameters in the solvent deasphalting process, such as the type and weight percentage of nanoadsorbents, and the volumetric ratio of solvent to fuel, in reducing the remaining sulfur content in Mazut fuel oil. We effectively utilized the CCD method to determine the optimal values for each treatment parameter by understanding the rules governing these factors. Additionally, CCD was employed to enhance the quadratic models for the response variables. Both models exhibited a strong fit (with R^2^ values of 0.9666 and 0.9665) indicating successful regression modeling. According to the results obtained from CCD/RSM, the lowest weight percentage of sulfur in DAO (2.46) was obtained under conditions where 2.5% of Silanated-Fe_2_O_3_ nanoadsorbent was used with a solvent to fuel volumetric ratio of 7.7 in the solvent deasphalting process. Also, the minimum sulfur content in DAO was attained at optimum condition of weight percentage of nanoadsorbent: 2.581, solvent to oil ratio: 7.704 and type of nanoadsorbent is Silanated-Fe_2_O_3_. The validity of the final model under optimal variable conditions was also confirmed by comparing the remaining sulfur values in DAO and laboratory conditions (2.455 and 2.41, respectively). Moreover, when the temperature rises to 70 °C and utilizing a nano-adsorbent of silanated-Fe_2_O_3_, the weight percentage of sulfur remaining in DAO dropped to 2.13%. This demonstrates a decrease of 39.14% in comparison to the original fuel. This study indicates that the nano-adsorbents has a significant impact on reducing the sulfur content in DAO specially when exposed to higher temperatures. This can be advantageous as high sulfur content in fuels can lead to increased emissions of sulfur dioxide, which is a major contributor to air pollution and acid rain. By using the nano-adsorbent, the sulfur emissions can be significantly reduced, promoting cleaner and more environmentally friendly combustion of the fuel.

### Supplementary Information

Supplementary Tables.

## Data Availability

All data generated or analyzed during this study are included in this published article and its supplementary information files.
