# Supplementary material for: Reduction of sulfur in fuel oil using Fe2O3 hybrid nanoadsorbent by solvent deasphalting and optimization of operational parameters with CCD
Source: Sci Rep. 2024 Jan 18;14:1560. doi: 10.1038/s41598-024-52166-5 (PMC10796671; doi:10.1038/s41598-024-52166-5)
Supplement: Supplementary file 1 — Supplementary Tables. [file 41598_2024_52166_MOESM1_ESM.pdf]

Supplementary file:

Reduction of Sulfur in Fuel oil Using Fe_2O_3 Hybrid Nanoadsorbent by Solvent Deasphalting and Optimization of Operational Parameters with CCD

Mohammadreza Malek[1], Mohammad Samipourgiri*[1], Alimorad Rashidi[2], Nasrolah Majidian[1]

[1]Chemical Engineering Department, Faculty of Engineering, North Tehran Branch, Islamic Azad University, Tehran, 1651153311, Iran
[2]Carbon & Nanotechnology Research Center, Research Institute of Petroleum Industry (RIPI), Tehran, 14857-33111, Iran
Corresponding Author: M_samipoor@iau-tnb.ac.ir

Table S1. ANOVA results for the sulfur content in DAO

Source	Sum of Squares	degree of freedom	Mean Square	F-value	p-value
Model	1.04	17	0.0613	35.70	< 0.0001
A-nano conc.	0.0241	1	0.0241	14.03	0.0012
B-solvent to oil ratio	0.0758	1	0.0758	44.17	< 0.0001
C-nano type	0.7481	2	0.3740	217.92	< 0.0001
AB	0.0114	1	0.0114	6.65	0.0175
AC	0.0317	2	0.0159	9.25	0.0013
BC	0.0083	2	0.0042	2.43	0.1127
A^2	0.0227	1	0.0227	13.24	0.0015
B^2	0.0850	1	0.0850	49.55	< 0.0001
ABC	0.0035	2	0.0017	1.01	0.3813
A^2C	0.0004	2	0.0002	0.1188	0.8886
B^2C	0.0394	2	0.0197	11.49	0.0004
Residual	0.0360	21	0.0017		
Lack of Fit	0.0354	9	0.0039	78.76	< 0.0001
Pure Error	0.0006	12	0.0001		
Total	1.08	38			

Table S2. ANOVA results for the pitch yield

Source	Sum of Squares	degree of freedom	Mean Square	F-value	p-value
Model	678.64	17	39.92	34.60	< 0.0001
A-nano conc.	56.41	1	56.41	48.90	< 0.0001
B-solvent to oil ratio	82.45	1	82.45	71.46	< 0.0001
C-nano type	344.37	2	172.19	149.24	< 0.0001
AB	2.00	1	2.00	1.73	0.2021
AC	57.16	2	28.58	24.77	< 0.0001
BC	29.25	2	14.63	12.68	0.0002
A^2	38.93	1	38.93	33.74	< 0.0001
B^2	64.46	1	64.46	55.87	< 0.0001
ABC	2.23	2	1.12	0.9671	0.3965
A^2C	12.75	2	6.37	5.52	0.0118
B^2C	0.0337	2	0.0168	0.0146	0.9855
Residual	24.23	21	1.15		
Lack of Fit	24.10	9	2.68	251.06	< 0.0001
Pure Error	0.1280	12	0.0107		
Cor Total	702.87	38			
